# The association between chemosensitivity and Pgp, GST-π and Topo II expression in gastric cancer

**DOI:** 10.1186/1746-1596-8-198

**Published:** 2013-12-10

**Authors:** Ming Geng, Lin Wang, Xin Chen, Ruixue Cao, Peifeng Li

**Affiliations:** 1 Department of Pathology, General Hospital of Jinan Military Command, Jinan, China; 2 Department of Laboratory Diagnosis, General Hospital of Jinan Military Command, Jinan, China

**Keywords:** Gastric cancer, MTT colorimetric assay, Drug sensitivity, Pgp, GST-π, Topo II

## Abstract

**Background:**

To investigate the relationship between P-glycoprotein (Pgp), glutathione S-transferase π (GST-π) and topoisomerase II (Topo II) expression and human gastric cancer chemoresistance *in vitro*.

**Methods:**

Primary single-cell suspensions were prepared from fresh specimens of primary gastric cancer and exposed to hydroxycamptothecin (HCPT), cisplatin (CDDP), 5-fluorouracil (5-FU), adriamycin (ADM) and mitomycin (MMC) for 48 h. Cell metabolic activity and rate of inhibition were evaluated using tetrazolium (MTT) assay. Pgp, GST-π and Topo II expression was determined in gastric carcinoma tissue samples using immunohistochemistry.

**Results:**

Chemosensitivity of the gastric cancer cells varied; the rates of inhibition of cells exposed to HCPT, CDDP and 5-FU were significantly higher than that of cells exposed to ADM and MMC (*p* < 0.05). Gastric cancer cells with Pgp expression were resistant to ADM and HCPT (*p* = 0.008 and *p* = 0.011, respectively). Cells resistant to 5-FU, CDDP and MMC had significantly higher GST-π expression (*p* < 0.05). Topo II expression was significantly lower in cells resistant to HCPT, ADM and MMC (*p* < 0.05). Pgp and GST-π expression may contribute to primary resistance of gastric cancer cells to some chemotherapeutic drugs, while Topo II expression may indicate HCPT, ADM and MMC sensitivity.

**Conclusions:**

Pgp, GST-π and Topo II detection and the MTT assay could be used as predictors in chemotherapeutic drug administration and for identifying drug resistance in gastric carcinoma.

**Virtual slides:**

The virtual slide(s) for this article can be found here: http://www.diagnosticpathology.diagnomx.eu/vs/3448329111142964.

## Introduction

Chemotherapy is an important element of systematic treatment of malignant tumors, while the main obstacle to effective chemotherapy is multidrug resistance (MDR). There are two major mechanism of MDR. The first and most important is transporter-based MDR caused by the activation of transporter proteins such as P-glycoprotein (Pgp) [1,2]; The second is non-transporter-based MDR, which is caused by altered activity of enzyme systems such as glutathione S-transferase π (GST-π), resulting in drug sequestration in intracellular vesicles [3]. Reduced expression of topoisomerase II (Topo II) in cancer tissue was closely related to MDR [4]. In this study, we cultured primary gastric cancer cells from freshly resected gastric cancer specimens *in vitro* and assessed their sensitivity to hydroxycamptothecin (HCPT), adriamycin (ADM), cisplatin (CDDP), 5-fluorouracil (5-FU) and mitomycin (MMC) by tetrazolium (MTT) colorimetric assay. Pgp, GST-π and Topo II expression were examined by immunohistochemical staining of paraffin-embedded gastric cancer tissue specimens. The relationship between chemoresistance and Pgp, GST-π and Topo II expression was explored to clarify the related factors of primary drug resistance in gastric cancer further.

## Materials and methods

### Specimen collection and preparation

The study included 81 patients with primary gastric cancer; it was approved by the General Hospital of Jinan Military Command Ethics Committee. The patients had undergone gastrectomy at the hospital from January 2007 to March 2009. After surgery, diagnosis was confirmed by pathology; tumor specimens without necrosis were collected for primary culture. Single-cell suspensions (1 × 10^5^ cells/mL) were prepared [5]. All patients provided written informed consent.

### MTT chemosensitivity assay

Gastric cancer cells were successfully cultured from 75 cases (93.75%). Aliquots (100 μL, 10^4^ cells) were plated into 96-well microplates (Gibco, Carlsbad, CA, USA). Drug solutions were dissolved in RPMI 1640 and 100-μL aliquots were added to each well to yield final concentrations of 0.3 μg/mL HCPT (Sanlian Co. Ltd., Heilongjiang, China), 3.0 μg/mL CDDP (Qilu Co. Ltd., Shandong, China), 1.0 μg/mL MMC (Huangshi Co. Ltd., Hubei, China), 50 μg/mL 5-FU (Hualian Co. Ltd., Shanghai, China), or 4 μg/mL ADM (Xinhua Co. Ltd., Shandong, China). Three duplicate wells were plated for each specimen. Control wells contained 100 μL cell suspension, 100 μL RPMI 1640 and 10% fetal bovine serum (FBS); 200 μL RPMI 1640 containing 10% FBS was used as the blank control. Microplates were incubated for 48 h at 37°C in a humidified atmosphere containing 5% CO_2;_ 20 μL 0.4% MTT (Sigma-Aldrich, St. Louis, MO, USA) and 0.1 M sodium succinate was added and the microplates were incubated for a further 4 h at 37°C. The optical densities of each well were determined using an SM-3 easy reader (Tianshi, Beijing, China) at 570 nm. The inhibition rates (IR) were calculated using the formula (A_c_–A_d_)/(A_c_–A_b_) × 100%, where A_d_, A_c_ and A_b_ represent the mean absorbance of drug-treated, control and blank wells, respectively. The results were defined as follows: highly sensitive, IR > 50%; moderately sensitive, IR 30%–50%; resistant, IR <30%.

### Pgp, GST-π and Topo II expression in gastric cancer

Immunohistochemical staining for Pgp, GST-π and Topo II was performed on formalin-fixed paraffin-embedded tissue sections of gastric cancer using the streptavidin-peroxidase method. All primary antibodies were purchased from Maixin Biotechnology Lnc (Fuzhou, China). The results were evaluated as previously described [6,7], i.e., by counting 100 cells per field in 10 random fields under high-magnification microscopy (×400, Olympus BX53 [Olympus, Tokyo, Japan]). Positive staining was defined as ≥25% staining; negative staining as <25% staining.

### Statistical analysis

Statistical analysis was carried out using SPSS v. 17.0 for Windows; *p*-values were two-sided, and *p* < 0.05 was considered significant. Quantitative results were expressed as mean ± standard error of the mean. Significant differences were determined using the *χ*^2^ test and rank sum test.

## Results

### Chemosensitivity assay

The MTT assay revealed that the drugs induced different levels of inhibition in the tumor cells (Table 1). The IR values in cells exposed to HCPT, CDDP and 5-FU were similar and significantly higher than that of cells exposed to ADM and MMC (rank sum test, *p* < 0.05).

**Table 1:** MTT assay of 75 cases of primary gastric cancer

**Drug**	**Sensitivity**			**Mean IR-**
	**High (%)**	**Moderate (%)**	**Low (%)**	
HCPT	7 (9.33)	25 (33.33)	43 (57.33)	40.6%^※^
CDDP	7 (9.39)	26 (34.67)	42 (56.00)	41.9%^※^
5-FU	9 (12.0)	29 (38.067)	37 (49.33)	43.4%^※^
ADM	4 (5.35)	27 (36.00)	44 (58.67)	31.6%
MMC	3 (4.00)	25 (33.33)	42 (62.67)	28.7%

^※^Compared with ADM and MMC: *P* < 0.05.

### Pgp, GST-π and Topo II expression in gastric cancer tissue

**Figure 1:**
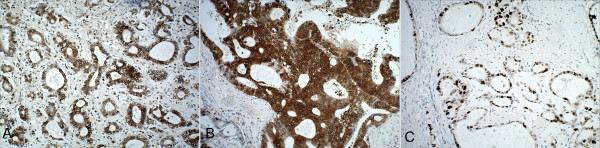
**Immunohistochemical findings in gastric cancer tissue. (A)** Positive Pgp staining in the cytoplasm and plasmalemma of tumor cells; **(B)** Positive GST-π staining in the cytoplasm of tumor cells; **(C)** Positive Topo II staining in the nuclei of tumor cells.

Pgp, GST-π and Topo II expression in gastric cancer was determined by immunohistochemical staining. Pgp expression was observed as brown-yellow particles in the cytoplasm and plasmalemma; GST-π and Topo II were visualized in the cytoplasm and nucleus, respectively (Figure 1). The rates of positive Pgp, GST-π and Topo II expression were 61.33% (46/75), 65.33% (49/75) and 68.00% (51/75), respectively. There was no statistical difference among the three proteins (*p* > 0.05).

### Relationship between Pgp, GST-π and Topo II expression and chemosensitivity

Tumor cells were considered chemosensitive if the IR was ≥30% and chemoresistant if the IR was <30%. Cells that expressed Pgp were resistant to HCPT and ADM (*p* < 0.05, Table 2), but not CDDP, 5-FU and MMC. Cells that expressed GST-π were resistant to CDDP, 5-FU and MMC (*p* < 0.05, Table 3). Topo II expression was related to sensitivity to HCPT, ADM and MMC (*p* < 0.05, Table 4).

**Table 2:** Relationship between Pgp expression and chemosensitivity

**Drug**	**Chemosensitivity**	**Cases (n)**	**Pgp expression**		***χ***^**2**^	***P***
			**Positive(n)**	**Negative(n)**		
HCPT	Sensitive	32	15	17	6.271	0.011
	Resistant	43	31	12		
CDDP	Sensitive	33	20	13	0.722	0.641
	Resistant	42	26	16		
5-FU	Sensitive	38	22	16	0.629	0.824
	Resistant	37	24	13		
ADM	Sensitive	31	11	20	9.350	0.008
	Resistant	44	35	9		
MMC	Sensitive	28	17	11	0.883	0.527
	Resistant	47	29	18		

**Table 3:** Relationship between GST-π expression and chemosensitivity

**Drug**	**Chemosensitivity**	**Cases (n)**	**GST-π expression**		***χ***^**2**^	***P***
			**Positive(n)**	**Negative(n)**		
HCPT	Sensitive	32	20	12	0.725	0.632
	Resistant	43	29	14		
CDDP	Sensitive	33	15	18	8.122	0.013
	Resistant	42	34	8		
5-FU	Sensitive	38	21	17	6.128	0.027
	Resistant	37	28	9		
ADM	Sensitive	31	20	11	0.850	0.578
	Resistant	44	29	15		
MMC	Sensitive	28	14	14	5.783	0.036
	Resistant	47	35	12		

**Table 4:** Relationship between Topo II expression and chemosensitivity

**Drug**	**Chemosensitivity**	**Cases (n)**	**Topo II expression**		***χ***^**2**^	***P***
			**Positive(n)**	**Negative(n)**		
HCPT	Sensitive	32	27	5	9.224	0.009
	Resistant	43	24	19		
CDDP	Sensitive	33	23	10	0.787	0.635
	Resistant	42	28	14		
5-FU	Sensitive	38	29	9	2.326	0.149
	Resistant	37	26	11		
ADM	Sensitive	31	26	5	7.250	0.015
	Resistant	44	25	19		
MMC	Sensitive	28	25	3	6.843	0.019
	Resistant	47	26	21		

## Discussion

Gastric cancer is one of the most common malignant tumors worldwide [8]. Despite effective control of the primary tumor and the availability of both neoadjuvant and adjuvant chemotherapy, it is currently the second leading cause of cancer death worldwide [9]. The poor prognosis is associated with extensive local invasion, regional lymph node metastasis, and chemoresistance [10]. Many cancer cells develop intrinsic and acquired resistance against chemotherapeutic agents structurally and mechanistically, thus chemotherapeutic complete response cannot be obtained for the majority of malignant tumors. Therefore, more studies on chemosensitivity and chemoresistance have focused on various transporter proteins inside tumor cells. MDR in tumor cells is generally considered the major factor of chemotherapy failure in patients with cancer [11,12]. In particular, overexpression or increased activity of the genes for Pgp and GST-π and low Topo II expression are closely associated with chemoresistance in many tumors. These proteins are involved in chemoresistance via many mechanisms, including increased drug efflux, decreased drug influx, drug inactivation and drug target alteration [13].

Encoded by the MDR 1 (*MDR1*) gene and located on 7q21.1, Pgp is a cell membrane-bound adenosine triphosphate-binding cassette transporter that actively extrudes a variety of chemotherapeutic drugs from cancer cells [1], thereby possibly being responsible for intrinsic and acquired drug resistance in numerous human cancers.By pumping lipophilic drugs out of cells, Pgp reduces intracellular drug concentrations and leads to drug resistance [14]. Triller and coworkers found that in 17 chemotherapy-naive small cell lung cancer patients, chemotherapy response was strongly associated with the level of Pgp expression [15]. Low Pgp expression was associated with good chemotherapy response, whereas higher expression predicted a worse outcome. Our study indicated that there was Pgp overexpression before chemotherapy in some gastric cancer cases and that it was a participant and mediator of gastric cancer cell resistance to ADM and HCPT. ADM and HCPT are anthracycline and alkaloid anti-cancer drugs, respectively; both are lipophilic drugs. Pgp-positive gastric cancer cells exhibited obvious resistance to ADM and HCPT, indicating that Pgp-positive gastric cancer is likelier to be resistant to HCPT and ADM. We believe that Pgp-related resistance mainly acts against natural and lipophilic anti-cancer drugs, which is consistent with the speculation of Pakos and Ioannidis [16]. Therefore, ADM and HCPT should not be recommended to Pgp positive gastric cancer patients; rather, alkylating agents and anti-metabolic drugs to which resistance is not closely related with Pgp expression, would be more appropriate.

GST-π, a member of the GST family, is a multifunctional enzyme that plays a critical role in cellular detoxification by catalyzing the conjugation of reduced glutathione to hydrophobic electrophilic compounds and may influence mutagenesis and carcinogenesis. GST-π overexpression has been observed in many tumors as compared to the surrounding normal tissues and in various cancer cell lines resistant to anti-cancer agents; GST-π has been used in cancer research as a tumor biomarker [17]. Soh *et al.* found that nuclear localization of GST-π was associated with both inherent and acquired drug resistance in gynecological cancers, which indicated that GST-π in malignant cells may be a useful predictor and may contribute to anti-cancer drug selection [18]. In our study, there was an obvious association between GST-π expression and resistance to antibiotics (MMC), metal anti-cancer drugs (CDDP) and 5-FU in chemotherapy-naïve patients, indicating that chemoresistance might occur in GST-π–positive gastric cancer. Based on the mechanism of resistance, we hypothesize that GST-π in combination with chemotherapy drugs and drug detoxification may play a major role in early resistance: higher GST-π expression, indicates lower cytotoxic effects of chemotherapy drugs, leading to tumor cell chemoresistance.

Topoisomerases are nuclear enzymes that play a key role in DNA replication. Topo II localization in the nucleus is involved in DNA transcription, translation and replication. It can mediate DNA cleavage and the formation of DNA enzyme complexes during the S-G_2_/M phase, which is an important target for a variety of chemotherapy drugs. It is mainly expressed during the S-phase and appears to be the preferred target associated with drug resistance [19]. The mechanisms of Topo II resistance are obviously different from that of Pgp and GST-π, and reduction of its expression or alteration of its properties would affect cross-linked DNA complex formation and reduce chemosensitivity. In our study, Topo II expression was significantly negatively correlated with HCPT, ADM and MMC resistance, suggesting that it is mainly involved in resistance to natural or semi-natural and antibiotic anti-cancer drugs, indicating the likelihood that Topo II negative gastric cancer would be resistant to HCPT, ADM and MMC. Previously, we demonstrated that the sensitivity of gastric cancer cells to some chemotherapy drugs was associated with histopathological type [5], suggesting that there is a greater proportion of proliferative-phase (S-G_2_/M phase) cells in poorly differentiated gastric cancer. In this phase, Topo II expression is increased, therefore there would be a high level of chemosensitivity to some of the drug effects in proliferating cells. This is related to the relative clinical sensitivity of poorly differentiated gastric cancer to some chemotherapy drugs.

In conclusion, Pgp, GST-π and Topo II expression differed in gastric cancer, and the difference may be associated with the variation in sensitivity to HCPT, CDDP, MMC, 5-FU and ADM. Pgp may be useful for predicting intrinsic resistance to HCPT and ADM; GST-π for CDDP, 5-FU and MMC resistance; and Topo II may be useful for predicting HCPT, ADM and MMC sensitivity. MDR might be simultaneously involved in the participation of multiple genes and molecular pathways. Combined identification of Pgp, GST-π and Topo II expression status may be valuable for screening the most appropriate low-toxicity and high-efficacy drugs prior to (neo)adjuvant chemotherapy and optimizing the most effective individualized chemotherapy regimens based on molecular biological mechanisms.

## Competing interests

The authors declare that they have no competing interests.

## Authors’ contributions

MG, LW and PFL carried out all evaluations and drafted the manuscript. LW and XC performed the MTT chemosensitivity assay and immunohistochemical examination. RXC collected the clinical data. PFL, MG and LW contributed to the conception and design of the study. All authors read and approved the final manuscript.
